# New approaches for the reliable in vitro assessment of binding affinity based on high-resolution real-time data acquisition of radioligand-receptor binding kinetics

**DOI:** 10.1186/s13550-016-0249-9

**Published:** 2017-03-07

**Authors:** Markus Zeilinger, Florian Pichler, Lukas Nics, Wolfgang Wadsak, Helmut Spreitzer, Marcus Hacker, Markus Mitterhauser

**Affiliations:** 1 Department of Biomedical Imaging and Image-guided Therapy, Division of Nuclear Medicine, Radiopharmacy and Experimental Nuclear Medicine, Waehringer Guertel 18-20, 1090 Vienna, Austria; 2 Faculty of Engineering, University of Applied Sciences Wiener Neustadt, Wiener Neustadt, Austria; 3 Department of Inorganic Chemistry, University of Vienna, Vienna, Austria; 4 Department of Pharmaceutical Chemistry, University of Vienna, Vienna, Austria; 5 Department of Pharmaceutical Technology and Biopharmaceutics, University of Vienna, Vienna, Austria; 6 Ludwig Boltzmann Institute for Applied Diagnostics, Vienna, Austria

**Keywords:** Real-time cell-binding studies, Kinetic competition assay, Binding kinetics, Binding affinity, Cell-based ligand interaction analysis, A_3_R

## Abstract

**Background:**

Resolving the kinetic mechanisms of biomolecular interactions have become increasingly important in early-phase drug development. Since traditional in vitro methods belong to dose-dependent assessments, binding kinetics is usually overlooked. The present study aimed at the establishment of two novel experimental approaches for the assessment of binding affinity of both, radiolabelled and non-labelled compounds targeting the A_3_R, based on high-resolution real-time data acquisition of radioligand-receptor binding kinetics. A novel time-resolved competition assay was developed and applied to determine the K_i_ of eight different A_3_R antagonists, using CHO-K1 cells stably expressing the hA_3_R. In addition, a new kinetic real-time cell-binding approach was established to quantify the rate constants *k*_on_ and *k*_off_, as well as the dedicated *K*_*d*_ of the A_3_R agonist [^125^I]-AB-MECA. Furthermore, lipophilicity measurements were conducted to control influences due to physicochemical properties of the used compounds.

**Results:**

Two novel real-time cell-binding approaches were successfully developed and established. Both experimental procedures were found to visualize the kinetic binding characteristics with high spatial and temporal resolution, resulting in reliable affinity values, which are in good agreement with values previously reported with traditional methods. Taking into account the lipophilicity of the A_3_R antagonists, no influences on the experimental performance and the resulting affinity were investigated.

**Conclusions:**

Both kinetic binding approaches comprise tracer administration and subsequent binding to living cells, expressing the dedicated target protein. Therefore, the experiments resemble better the true in vivo physiological conditions and provide important markers of cellular feedback and biological response.

## Background

Binding studies on pharmacological targets are undoubtedly an indispensable part in drug development, to determine pharmacokinetic parameters of biomolecular interactions. The reliable in vitro assessment of ligand affinity, as well as the understanding of the binding process itself, provides an important contribution to the concept of selective targeting particular bioactive macromolecules in modern molecular medicine. During early-phase drug development, typically, such in vitro studies are conducted under closed-system conditions as saturation and competition experiments, which provide quantitative parameters for the extent of compound binding to a target region (e.g. receptor) according to the law of mass action [1]. In a conventional competitive binding experiment, the binding of one fixed concentration of a known radioligand is measured at equilibrium in the presence of a stepwise increasing series of concentrations of a non-labelled ligand. Data thus obtained are used to quantify the half-maximum inhibitory concentration (IC_50_) of the non-labelled ligand [2]. Subsequently, the equilibrium inhibitory constant (*K*_*i*_) can be calculated using the Cheng-Prusoff transformation [3], which serves as an indirect parameter for target affinity of the non-labelled ligand.

In contrast, the binding at equilibrium of an increasing series of concentrations of a radioligand to a particular target region is measured in a classical saturation experiment in order to quantify the equilibrium dissociation constant (*K*_*d*_) and the concentration of specific binding sites (*B*_Max_) for the radioligand [4]. Since both traditional in vitro methods belong to dose-dependent assessments, ligand-receptor binding kinetics is usually overlooked. Resolving the kinetic mechanisms of biomolecular interactions governing ligand association and dissociation has become more and more important to improve the performance of binding experiments. Several lines of research retrospectively suggested that high temporal information about the binding kinetics can assist to avoid systematic bias and potential errors of obtained data under equilibrium and non-equilibrium conditions [5–7]. Furthermore, the validation and interpretation of time-resolved pharmacokinetic data advance the formulation of computational methods to analyse biomolecular interactions of ligands with the receptor alone, or even in combination.

In contrast to in vitro binding studies, in an in vivo setting, the concentration of a dedicated ligand to its target region is no longer constant, but changes over time after administration is often influenced by additional factors other than basic biomolecular ligand-receptor interactions. Therefore, the in vitro measured K_d_ alone is not anymore an informative parameter to characterize complex compound interactions, as well as in vivo effectiveness of small molecule drugs, but rather the in vitro assessment of kinetic parameters such as the association rate constant (*k*_on_) and the dissociation rate constant (*k*_off_) [8, 9]. As a consequence, various experimental procedures have been introduced to address a shift from classical affinity-based assessments to kinetic binding approaches [5, 6, 10–16]. In this context, the usual practice of performing kinetic binding experiments is to measure the binding of one or more concentrations of a radioligand with low nanomolar affinity dedicated to the target at various time points and determine *k*_on_ and *k*_off_. With both rate constants, *K*_*d*_ can be calculated as the ratio of *k*_off_/*k*_on_ [12]. However, the currently available experimental techniques for the determination of kinetic binding parameters are time-consuming, laborious and lack the possibility of data acquisition with high temporal resolution. For that reason, the existing methods are prone to errors and reduce the scale of applications in determining biomolecular interactions, as well as hamper the high-throughput screening of important binding properties in early-phase drug discovery [15, 17, 18].

In the present study, we introduce two experimental approaches for the reliable high-throughput in vitro assessment of binding parameters of both, radiolabelled and non-labelled compounds, based on high-resolution, real-time data acquisition of radioligand-receptor binding kinetics. Herein, we present in competitive real-time cell-binding studies an experimental assay to determine the IC_50_ out of kinetic data in order to calculate the dedicated *K*_*i*_ of a non-labelled ligand. Although previously published work already addressed determination of *k*_on_, *k*_off_, *K*_*d*_, *K*_*i*_ and *B*_Max_ [19–22], in the present kinetic real-time cell-binding studies, we established an alternative experimental design for the assessment of the rate constants *k*_on_ and *k*_off_. Therefore, the observed rate constant of the association reaction (*k*_ob_) is used to calculate these parameters out of different increasing concentrations of radioligand. The technical principle of the experimental procedures is based on an equipment technology called LigandTracer® (Ridgeview Instruments AB, Uppsala, Sweden), which was successfully established and implemented in a variety of studies dealing with pharmacokinetic aspects [23–26]. This technique comprises the possibility of automated high-resolution real-time quantification of biomolecular interactions by use of repeated differential measurements of bound radioligand on cell surface proteins [23]. As a result, we are capable of following the uptake, retention and dissociation of a radioligand to its dedicated target region directly during the experiment.

In the current study, we used the adenosine A_3_ receptor (A_3_R) as a sample target to validate the suitability of the used system and the experimental approaches. Adenosine is an important cell modulator and acts as an endogenous quieting substance via four subtypes of different G-protein-coupled receptors (GPCRs), termed A_1_, A_2A_, A_2B_ and A_3_ receptors [27, 28]. The A_3_R is a promising target for molecular imaging, since it is involved in many of the body’s cytoprotective functions and changes of the expression leads to a variety of pathologies, especially neurological and affective disorders, cardiac diseases, oncological diseases and inflammation processes [29–31]. In this context, the well-known and high affinity A_3_R agonist, [^125^I]-4-aminobenzyl-5′-*N*-methylcarboxamideoadenosine ([^125^I]-AB-MECA) was used to serve as radiolabelled reference compound for both experimental approaches [32, 33]. Commercially available A_3_R antagonists, 1,4-dihydro-2-methyl-6-phenyl-4-(phenylethynyl)-3,5-pyridinedicarboxylic acid, 3-ethyl 5-(phenylmethyl) ester (MRS1191) and 2,3-ethyl-4,5-dipropyl-6-phenylpyridine-3-thiocarboxylate-5-carboxylate (MRS1523) were used as non-labelled model compounds for the verification of the competitive real-time cell-binding assay [34–36]. In addition, six different in-house synthesized A_3_R antagonists [37–39] were screened in competitive real-time cell-binding studies in order to illustrate the feasibility of the used method. To avoid potential errors and confounding factors due to the physicochemical properties of the used compounds, commercially available and in-house synthesized A_3_R antagonists were subjected to lipophilicity measurements to determine the logarithm of the octanol-water partition coefficient.

## Methods

### Cell culture and petri dish preparation

Cell-binding studies were conducted on adherent Chinese hamster ovary cells (CHO-K1) and CHO-K1 cells stably expressing the human adenosine A_3_ receptor (CHO-K1-hA_3_R). Both cell lines were a generous gift from Professor Karl-Norbert Klotz (University of Würzburg, Germany) [40]. CHO-K1 and CHO-K1-hA_3_R cells were cultured in Ham’s F-12 medium (Gibco®, Life Technologies) containing 1% penicillin-streptomycin-glutamine (PSG), 10% fetal bovine serum (FBS) and 300 μg/mL Geneticin (G-418) and incubated in a humidified 5% CO_2_ atmosphere at 37 °C. Cells were grown up to 70% confluence in dedicated tissue culture flask (175 cm^3^, CELLSTAR®, Greiner Bio-One) and subcultured latest after 2 days to assure proper cell density, growth and differentiation. The splitting process was induced by washing the cells with 5 mL Dulbecco’s phosphate-buffered saline (DPBS) for 10 s followed by the incubation with 5 mL Trypsin-EDTA for 5 min. Subsequently, the cells were resuspended in 10-mL cell culture medium, transferred to a 50-mL conical centrifugation tube (Greiner Bio-One) and centrifuged at 200*g* (1000 rpm) for 5 min. The cell pellet was diluted with 1-mL medium and spread into a new cell culture flask containing 20 mL Ham’s F-12 medium with additives.

**Fig. 1:**
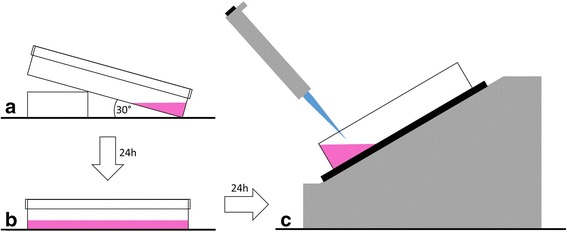
Schematic illustration of the petri dish preparation. 10^6^ CHO-K1-hA_3_R cells were seeded on a local part a tilted petri dish and incubated in 2 mL of medium for 24 h (**a**). On the consecutive day, the petri dish was preserved in a horizontal position and the cells were cultured with 10 mL of fresh medium for additional 24 h (**b**). On the third day, the petri dish was subjected to the binding experiment and the cells were provided with 3 mL of medium without additives (**c**)

Three days prior to a binding experiment with LigandTracer®, petri dishes with approximately 10^6^ cells were prepared (Fig. 1). On the first day, the cells were seeded as a monolayer on a local part on the bottom of a tilted cell culture dish (100 mm × 20 mm, CELLSTAR®, Greiner Bio-One) and incubated with 2-mL medium for 24 h in order to avoid cell migration. On the consecutive day, the old medium was discarded and the petri dish was preserved in a horizontal position with 10-mL medium for additional 24 h. On the third day, the binding experiment was performed, with fresh medium (3 mL Ham’s F-12, serum free).

### Radioligands and chemical compounds

The radioionated no-carrier-added (n.c.a.) A_3_R agonist [^125^I]-4-aminobenzyl-5′-*N*-methylcarboxamideoadenosine ([^125^I]-AB-MECA) was purchased from PerkinElmer® (PerkinElmer, Inc., Waltham, USA). [^125^I]-AB-MECA was described to reveal high affinity (K_d_ = 0.59 nM) towards the A_3_R and used to serve as radiolabelled reference compound with high specific activity (81.4 TBq mmol^−1^) for both experimental approaches [32, 33].

A_3_R antagonists, MRS1191 and MRS1523 were purchased from Sigma-Aldrich (Sigma-Aldrich St. Louis, MO, USA). The pentasubstituted pyridine derivatives 5-(2-fluoroethyl)2,4-diethyl-3-(ethylsulfanylcarbonyl)-6-phenylpyridine-5-carboxylate (FE@SUPPY), 5-ethyl 2,4-diethyl-3-((2-fluoroethyl)sulfanylcarbonyl)-6-phenylpyridine-5-carboxylate (FE@SUPPY:2), 4,6-diethyl-5-[(ethylsulfanyl)carbonyl]-2-phenylpyridine-3-carboxylic acid (DFE@SUPPY), methyl 4,6-diethyl-5-{[(2-fluoroethyl)sulfanyl]carbonyl}-2-phenylpyridine-3-carboxylate (FEMe@SUPPY:2), 2-fluoroethyl 4,6-diethyl-5-{[(2-fluoroethyl)sulfanyl]carbonyl}-2-phenylpyridine-3-carboxylate ((FE)^2^@SUPPY) and methyl 4,6-diethyl-5-[(methylsulfanyl)carbonyl]-2-phenylpyridine-3-carboxylate ((Me)^2^@SUPPY) were synthesized in our department as described previously [37–39]. All other chemicals were of analytical grade and purchased from commercial sources.

### Measurements of the logarithm of the octanol-water partition coefficient

The logarithm of the octanol-water partition coefficient of the A_3_R antagonists were determined at pH 7.4 by high-performance liquid chromatography (HPLC)-based assay (_HPLC_logD^7.4^) modified from Donovan and Pescatore [41]. Briefly, a mixture of two internal standards (toluene and triphenylene) with known logD values and defined retention times (k’) have been used. A_3_R antagonists were dissolved in methanol and injected onto a short polymeric ODP-50 column (20 × 4.0 mm, 5 μm pore size, Supelco, Bellefonte, PA, USA). A linear gradient from 10% methanol/90% phosphate buffer (25 mM, pH 7.4) to 100% methanol within 9.4 min at a flow rate of 1.5 mL/min were applied. Measurement and detection of the three retention times of toluene, triphenylene and A_3_R antagonists were performed at 260 and 285 nm. Between consecutive HPLC runs, 5 min for re-equilibration of the HPLC column were allowed. The logD values of the dedicated compounds were determined using the known logD values of triphenylene and toluene and the retention time of triphenylene, toluene and the unknown compounds according to the following formula:

1 $$ \log {P}_{\mathrm{Unknown}}=\frac{\left( \log {P}_{\mathrm{tol}}- \log {P}_{\mathrm{triph}}\right)*{t}_{\mathrm{Unknown}}+{t}_{\mathrm{tol}}* \log {P}_{\mathrm{triph}}-{t}_{\mathrm{triph}}* \log {P}_{\mathrm{tol}}}{t_{\mathrm{tol}}-{t}_{\mathrm{triph}}} $$

where “log *P*_Unknown_” is the logD value of the test compound and “*t*_Unknown_” the corresponding measured retention time of the dedicated substance. The logD values for the internal standards are abbreviated as “log *P*_tol_” (toluene) and “log *P*_triph_” (triphenylene), respectively. Measured retention times are indicated as “*t*_tol_” for toluene and “*t*_triph_” for triphenylene.

### Kinetic real-time cell-binding studies

**Fig. 2:**
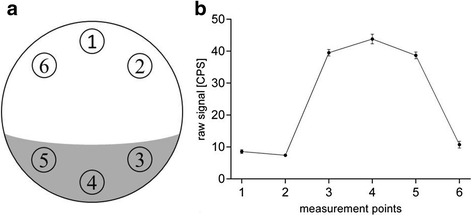
Representation of the measurement protocol. Real-time binding data of both experimental approaches were acquired according to repeated measurements of six dedicated reading points on a rotating petri dish (**a**). Measured points 1, 2 and 6 were located in the cell-free area (reference pole), whereas 3, 4 and 5 were set on the area in which the cells were seeded (cell pole). Data from each reading point were acquired over 4 s with a delay of 2 s between successive measurements. The corresponding radioactive signal with respect to the dedicated measurement point was monitored continuously in the perimeter trace (**b**). Representative graph from one experiment. Data are shown as mean ± SEM from eight consecutive measurements at binding equilibrium. If not visible, error bars are within the margin of the symbols

The assay protocol comprised repeated measurements of three points of the dedicated area in which the cells were seeded (cell pole) and three points of the opposing cell-free area (reference pole) of the petri dish as shown in Fig. 2a. Target distribution and cell attachment of the CHO-K1-hA_3_R cells were monitored continuously during the whole course of the binding experiment in the perimeter trace (Fig. 2b). Raw signal levels from consecutive measurements (*n* = 8) of a representative experiment after binding equilibrium of 0.7 nM [^125^I]-AB-MECA were clearly distinguishable and determined to be 39.51 ± 0.50, 43.82 ± 0.76 and 38.69 ± 0.52 for the cell pole and 8.55 ± 0.29, 7.41 ± 0.22 and 10.74 ± 0.52 for the reference pole, respectively, which is outlined in Fig. 2b. The corresponding binding trace of the radioligand was calculated as the difference between the averaged values of the cell pole and the reference pole yielding in a stable background-corrected signal. Kinetic real-time cell-binding studies were conducted on CHO-K1-hA_3_R cells with [^125^I]-AB-MECA at ambient temperature with LigandTracer® Grey. Repeated measurements of the cell pole and the reference pole of the dedicated petri dishes were performed as shown in Fig. 2a. The experiments were started with baseline measurements 10–15 min prior radioligand incubation. Binding of [^125^I]-AB-MECA to the seeded cells was realized by adding different concentrations (0.05–5 nM) to fully cover the concentration span needed for proper affinity estimation. Each dedicated concentration was incubated separately in a single petri dish, and the observed rate constants of the association reaction (k_obs_) were monitored in real-time until binding equilibrium. Unspecific uptake of the radioligand on the cell surface was confirmed with native CHO-K1 cells. As mentioned previously, the perimeter trace of the LigandTracer® was used to assure cell viability and attachment.

### Competitive real-time cell-binding studies

The measurements were made according to the assay protocol as previously described above. Competitive real-time cell-binding studies were performed on CHO-K1 (negative control) and CHO-K1-hA_3_R cells in presence of 0.7 nM [^125^I]-AB-MECA at ambient temperature with LigandTracer® Grey (Ridgeview Instruments AB, Uppsala, Sweden). The experiments were initiated with a baseline measurement (10–15 min). Subsequent binding association of [^125^I]-AB-MECA was introduced and continuously observed until binding equilibrium (60–90 min). Inhibition of the radioligand was performed by adding different concentration series (1–500 nM) of unlabelled compounds (A_3_R antagonists) each after visual binding equilibrium. At least four different concentrations of competitor, spanning three orders of magnitude adjusted appropriately for the IC_50_ of each compound, were used. Unspecific binding was determined in the presence of a high concentration (0.5–100 μM) of the non-labelled competitor substance. The observed association phase of the radioligand and the dissociation phase of each consecutive concentration of the competitor substance were monitored in real-time until visual binding equilibrium was reached. All non-labelled compounds were initially dissolved in DMSO and diluted with deionized water to the final concentration, where the amount of DMSO never exceeded 5%. Cytophysiological conditions and cell attachment were examined continuously during the whole course of the experiment with the perimeter trace of the LigandTracer® software (version 1.0.1).

### Data analysis and statistical procedures

Raw data from the LigandTracer® measurements were analysed in GraphPad Prism 6.0 (GraphPad Software, Inc., San Diego, CA). Experiments were conducted at least as a triplicate with different batches of radioligand and biological target material to avoid systematic bias and to assure statistical certainty. In competitive real-time cell-binding studies binding association of [^125^I]-AB-MECA was transformed to start at time point zero. Transformed data was analysed using “one-phase association” curve fitting algorithm as outlined in the following equation:

2 $$ Y={Y}_0 + \left(\mathrm{Plateau}-{Y}_0\right)*\left(1-{e}^{-k*x}\right) $$

where “*x*” is a given time and “*Y*” corresponds to the amount of radioligand binding for a dedicated time point. “*Y*_0_” is the initial value of “*Y*” at the beginning of radioligand incubation and “plateau” indicates the maximum amount of the bound radioligand after equilibrium. “*k*” is the rate constant expressed in reciprocal units of “*x*”.

Observed dissociation phases of [^125^I]-AB-MECA governed by different concentrations of cold competitor were transformed to start at time point zero. Transformed data were analysed by using “dissociation—one-phase exponential decay” curve fit as follows:

3 $$ Y=\left({Y}_0-\mathrm{N}\mathrm{S}\right)*{e}^{-k*x} + \mathrm{N}\mathrm{S} $$

where “*x*” is a given time and “*Y*” corresponds to the amount of bound radioligand for a dedicated time point. “*Y*_0_” is the initial value of “*Y*” at the beginning of each dissociation phase and “NS” indicates the steady state of radioligand binding in the presence of dedicated cold competitor after infinite time. “*k*” is the rate constant expressed in reciprocal units of “*x*”.

Equilibrium binding data determined in Eqs. (2) and (3) served as input parameters for the calculation of the IC_50_ using “log(inhibitor) vs. response—variable slope (four parameters)” nonlinear regression algorithm as described below:

4 $$ Y=\mathrm{Bottom} + \left(\mathrm{Top}-\mathrm{Bottom}\right)/\left(1+{10}^{\left(\left( \log \left({\mathrm{IC}}_{50}\right)-C\right)*\mathrm{HillSlope}\right)}\right) $$

where “*C*” is the logarithm of the competitor concentration and “*Y*” reflects the dedicated amount of bound radioligand at equilibrium obtained from Eq. (3). “Top” indicates the maximum amount of radioligand binding (without any competitor) whereas the corresponding value is derived from Eq. (2). “Bottom” expresses the amount of unspecific radioligand binding in presence of a very high concentration of competitor. The dedicated value is determined by Eq. (3) and corresponds to the “NS” value of the last inhibitor concentration. The “HillSlope” reflects the slope factor, which indicates the steepness of the resulting inhibition curve. “log(IC_50_)” represents the logarithm of the competitor concentration where 50% of the initial amount of bound radioligand is inhibited. The resulting IC_50_ was converted into the *K*_*i*_ using Cheng-Prusoff Eq. (3).

Data of the observed association phases determined in kinetic real-time cell-binding studies were transformed to start at time point zero. Transformed data were grouped together and globally fitted by using “association kinetics—two or more conc. of hot” to derive a single best-fit estimate for *k*_on_ and *k*_off_ as described as follows:

5 $$ Y={Y}_{\max }*1 - {e}^{-{k}_{\mathrm{ob}}*x} $$

where “*x*” is a given time and “*Y*” reflects the amount of bound radioligand for a dedicated time point. “*Y*_max_” corresponds to the maximum amount of bound radioligand after equilibrium and “*k*_ob_” expresses the observed rate constant of the association reaction.

The observed rate constant of the association reaction is a function of the dedicated radioligand concentration, the association rate constant and the dissociation rate constant, which is described as follows:

6 $$ {k}_{\mathrm{ob}}={k}_{\mathrm{on}}*L+{k}_{\mathrm{off}} $$

where “*k*_ob_” is the observed rate constant of the association reaction, “*k*_on_” represents the association rate constant, “*L*” reflects the radioligand concentration and “*k*_off_” indicates the dissociation rate constant. Considering Eqs. (5) and (6), best fit estimations for *k*_on_ and *k*_off_ can be calculated by using multiple radioligand concentrations. The resulting rate constants were used to obtain the *K*_*d*_ as the ratio of *k*_off_/*k*_on_. An estimation of the target concentration of each single petri dish was derived by using the following equation:

7 $$ {Y}_{\max }=\left(L/\left(L+{K}_d\right)\right)*{B}_{\max } $$

where “*Y*_max_” is the maximum amount of bound radioligand after equilibrium and “*B*_max_” reflects the maximum amount of target receptors. “*L*” corresponds to the radioligand concentration and “*K*_*d*_” indicates the affinity of dedicated radioligand.

Unless mentioned otherwise, all experimental data are expressed as mean ± SEM as determined by GraphPad Prism 6 (GraphPad Software, Inc., San Diego, CA) software analysis. Descriptive statistical measures were used to confirm the goodness of the nonlinear regression models. Differences among groups were proved using two-tailed, unpaired Student’s *t* test with Welch’s correction. Multiple comparisons testing was performed using either ordinary one-way ANOVA with Tukey’s correction or ordinary two-way ANOVA with Sidak’s correction. Values of *P* < 0.05 were considered as statistically significant.

## Results

### Verification of the target distribution and hA_3_R expression

**Fig. 3:**
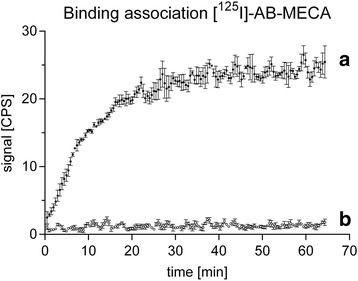
Representative association kinetics of [^125^I]-AB-MECA. Binding association of 0.7 nM [^125^I]-AB-MECA at ambient temperature to CHO-K1-hA_3_R cells (**a**) and to CHO-K1 cells, without the dedicated target receptor (**b**). Differences were proved to be statistically significant (*P* < 0.0001) using two-way repeated measures ANOVA with Sidak’s multiple comparisons test. Data are expressed as mean ± SEM from three independent experiments. If not visible, error bars are within the margin of the symbols

Kinetic studies with both CHO-K1 and CHO-K1-hA_3_R cells were conducted in a reliable and reproducible manner with high spatial and temporal resolution to verify specific radiotracer uptake on the dedicated target receptor (Fig. 3). Differences of the binding association of [^125^I]-AB-MECA between CHO-K1 and CHO-K1-hA3R from three independent experiments were proved to be statistically significant (*P* < 0.0001) using two-way repeated measures ANOVA with Sidak’s multiple comparisons test.

### Measurements of the logarithm of the octanol-water partition coefficient

Lipophilicity measurements were performed to evaluate the _HPLC_logD^7.4^ of the A_3_R antagonists, used in the competitive real-time cell-binding studies. Data were determined with high reliability and reproducibility and found to show considerable differences among the dedicated compounds ranging from 0.97 ± 0.37 up to 4.85 ± 0.01. A detailed summary of the corresponding _HPLC_logD^7.4^ values is shown in Table 1. Data are expressed as mean ± SEM of three independent experiments, each performed in triplicate.

**Table 1:**
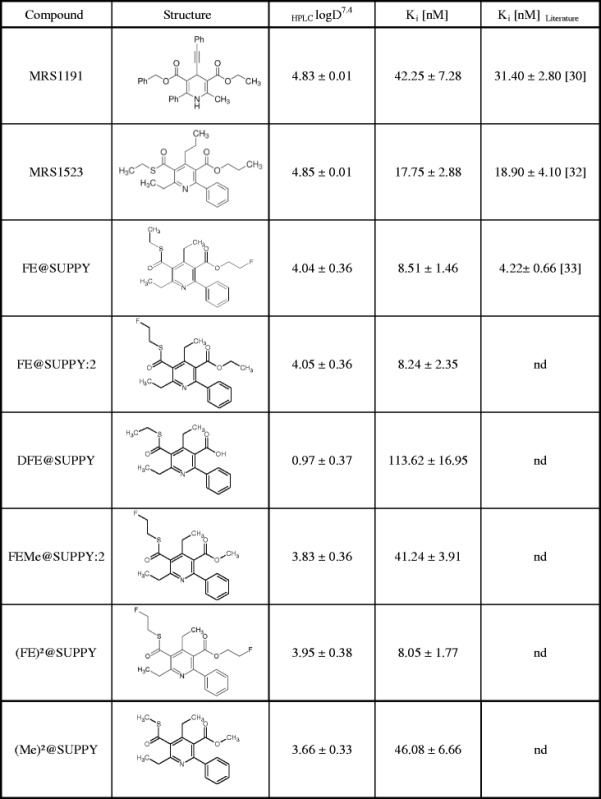
Estimates of the *K*_*i*_ values determined in competitive real-time cell-binding experiments

Displacement of specific [^125^I]-AB-MECA binding on CHO-K1-hA_3_R cells at ambient temperature in presence of different A_3_R antagonists with corresponding _HPLC_logD^7.4^ parameters. Reported affinity values were determined with traditional binding assays using membranes from human A_3_R-transfected HEK-293 cells. Data in the table are expressed as mean ± SEM from three independent experiments

*nd* not determined

### Competitive real-time cell-binding studies

**Fig. 4:**
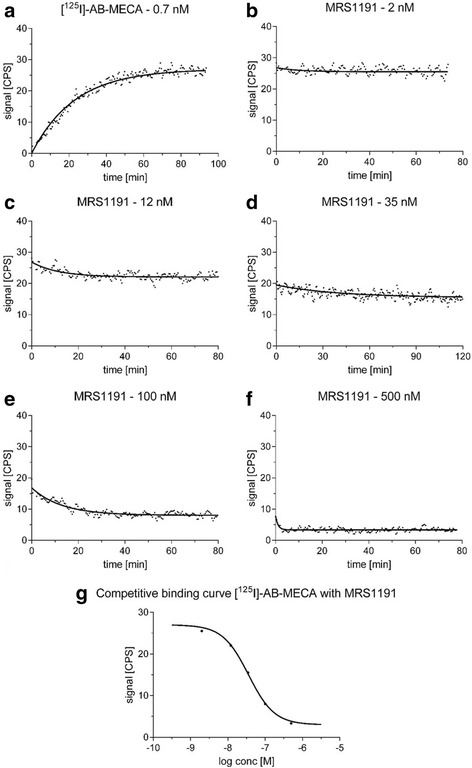
Schematic representation of the competitive real-time cell-binding experiment. Association kinetics of 0.7 nM [^125^I]-AB-MECA at ambient temperature to CHO-K1-hA_3_R (**a**). Association data were fitted in GraphPad Prism 6.0 using “one-phase association”. The value corresponding to the amount of bound radioligand at equilibrium was obtained using Eq. (2). Consecutive concentration-dependent dissociation kinetics of 0.7 nM [^125^I]-AB-MECA in the presence of 2 nM (**b**), 12 nM (**c**), 35 nM (**d**), 100 nM (**e**) and 500 nM (**f**) of the A_3_R antagonist MRS1191, respectively. Dissociation data were fitted using “dissociation—one-phase exponential decay”. Values of the remaining radioligand binding after equilibrium were obtained from Eq. (3). The competitive binding curve of [^125^I]-AB-MECA with MRS1191 (**g**) was created by means of combination of the equilibrium binding data obtained in Eqs. (2) and (3). Data were fitted using “log(inhibitor) vs. response—variable slope (four parameters)” nonlinear regression algorithm and corresponding IC_50_ values were obtained from Eq. (4). As different concentrations of competitor were used for each individual experiment, data in the graphs are shown from one representative experiment

**Fig. 5:**
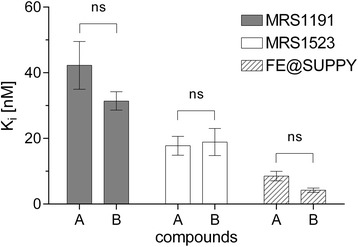
Comparison of obtained and reported *K*_*i*_ values. Inhibitory constants of the A_3_R antagonists MRS1191, MRS1523 and FE@SUPPY, respectively, were obtained from competitive real-time cell-binding studies at ambient temperature with 0.7 nM [^125^I]-AB-MECA on CHO-K1-hA_3_R cells (group A) and compared to the corresponding *K*_*i*_ values obtained from traditional binding assays, as previously reported in the literature (group B). Differences for MRS1191, MRS1523 and FE@SUPPY among groups are statistically not significant (ns = *P* > 0.05), using ordinary two-way ANOVA with Sidak’s correction. Data in the graph are shown as mean ± SEM from three independent experiments (see Table 1 for summary of dedicated *K*_*i*_ values)

An illustration of a representative competitive real-time cell-binding experiment with [^125^I]-AB-MECA is shown in Fig. 4. Binding kinetics of radioligand association and dissociation were obtained with high precision. As a result, the applied curve fitting algorithms were found to be highly accurate and revealed values for the coefficient of determination of 0.9503 ± 0.018 (association phases) and 0.8136 ± 0.087 (dissociation phases), respectively. Equilibrium binding data obtained from Eqs. (2) and (3) were proved to serve as stable input parameters for the generation of the competitive binding curve and the associated determination of the IC_50_. Displacement of specific [^125^I]-AB-MECA binding in the presence of different A_3_R antagonists revealed estimates for the *K*_*i*_ values as detailed in Table 1. Results from our experiments demonstrate that the inhibitory constants were varied with respect to modifications on the leaving groups of the pentasubstituted pyridines, whereas a high preservation of the chemical structure among compounds were proved to reveal in similar *K*_*i*_ values. Taking into account the lipophilicity of the tested A_3_R antagonists, no influences on the experimental performance and the resulting affinity were observed. Nevertheless, a slight trend was shown, which a decrease of the logD value is associated with an increasing *K*_*i*_. Differences of the inhibitory constants of MRS1191, MRS1523 and FE@SUPPY obtained from our competitive real-time cell-binding experiments and those previously reported with membranes from human A_3_R transfected HEK-293 cells in traditional binding assays [30, 32, 33] were found to be statistically not significant (*P* > 0.05) using ordinary two-way ANOVA with Sidak’s correction (Fig. 5).

### Kinetic real-time cell-binding studies

**Fig. 6:**
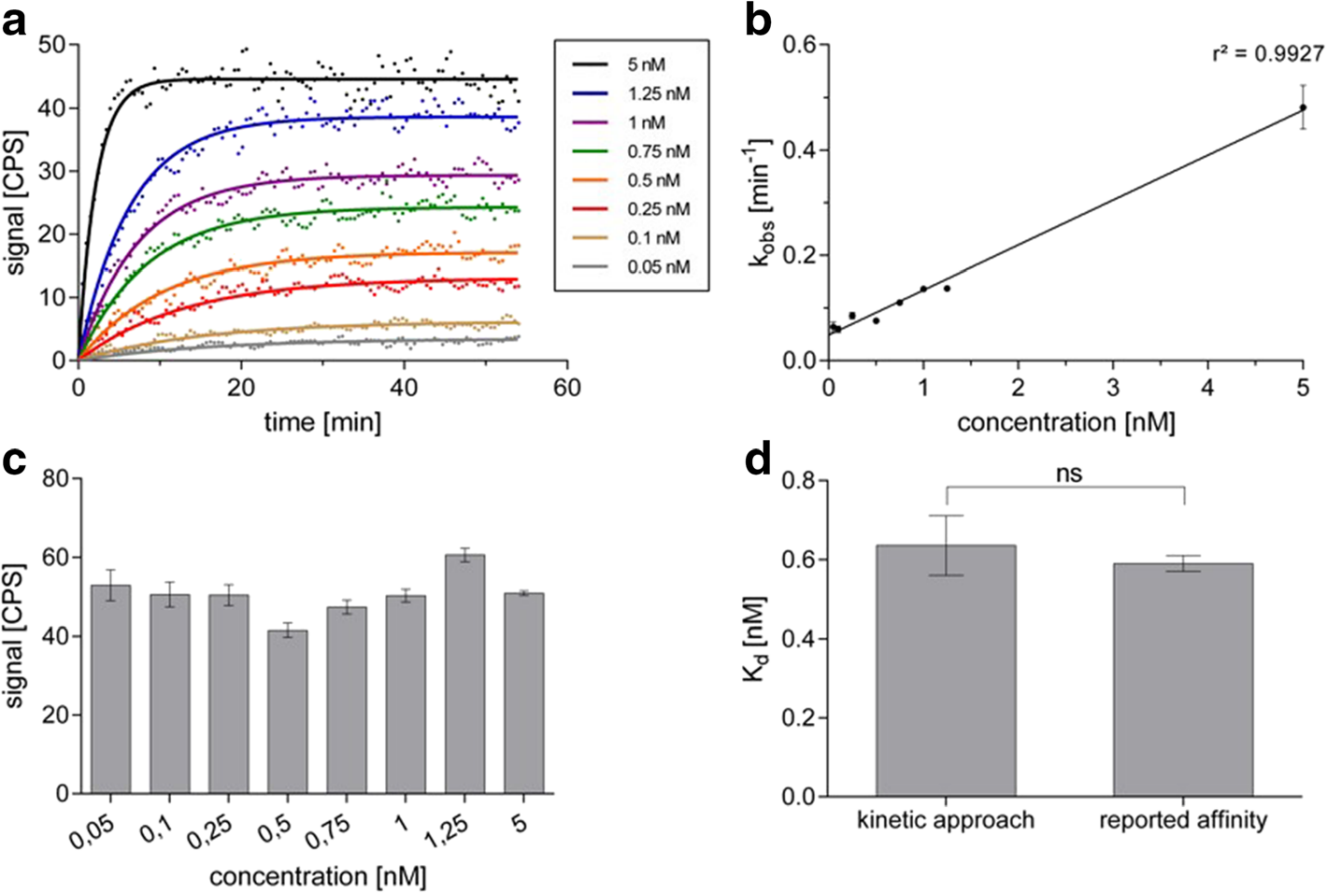
Graphical representation of the kinetic real-time cell-binding approach. Observed association time-courses of [^125^I]-AB-MECA to CHO-K1-hA_3_R at several concentrations (0.05–5 nM) spanning the *K*_*d*_ (**a**). Real-time binding data were globally fitted in GraphPad Prism 6.0 using “association kinetics—two or more conc. of hot” (*solid lines*). Best-fit estimations for *k*_on_ and *k*_off_ were obtained from Eqs. (5) and (6). Resulting rate constants were used to determine the *K*_*d*_ (see Table 2 for summary of dedicated *k*_on_, *k*_off_ and *K*_*d*_ values). To verify these results, *k*_obs_ values were determined using Eq. (2) and plotted against the corresponding concentration of [^125^I]-AB-MECA (**b**). Values for *k*_on_ and *k*_off_ calculated from linear regression analysis are in good agreement with values obtained from the global fit estimation. An estimation of the associated target concentration of each single petri dish was used to confirm homogenous receptor expression within the whole binding experiment (**c**). Values corresponding to the maximum amount of target receptors were obtained using Eq. (7). The *K*_*d*_ of [^125^I]-AB-MECA obtained from the novel kinetic real-time cell-binding studies and from traditional binding assays, as previously reported in the literature (see Table 2 for detailed summary of *K*_*d*_ values), was compared using two-tailed, unpaired Student’s *t* test with Welch’s correction (**d**). Differences between the K_d_ values are statistically not significant (ns = *P* > 0.05). Data in the graph (**a**) are shown from one representative experiment. Data in the graphs (**b**–**d**) are shown as mean ± SEM from individual experiments (*n* ≥ 4)

**Fig. 7:**
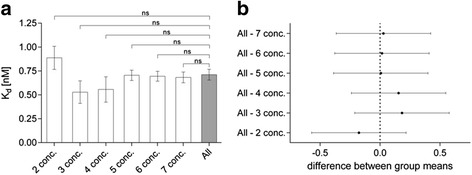
Verification and performance analysis of kinetic-real time cell-binding studies. Best-fit estimations for *k*_on_ and *k*_off_ were obtained using Eqs. (5) and (6), as detailed in the methods, and resulting *K*_*d*_ were calculated as the ratio of *k*_off_/*k*_on_. Fitted rate constants were determined using eight different concentrations (0.05–5 nM) of [^125^I]-AB-MECA to fully cover the concentration span needed for proper affinity estimation. The corresponding *K*_*d*_ (all) was compared to *K*_*d*_ values, calculated from best-fit estimations for the rate constants using two (2 conc.), three (3 conc.), four (4 conc.), five (5 conc.), six (6 conc.) or seven (7 conc.) concentrations of the radioligand, respectively (**a**). Differences between the *K*_*d*_ values are statistically not significant (ns = *P* > 0.05), using ordinary one-way ANOVA with Tukey’s multiple comparisons correction (see Table 2 for detailed summary of *k*_on_, *k*_off_ and *K*_*d*_ values). Differences between group means of the dedicated *K*_*d*_, calculated using the full set of concentrations and the *K*_*d*_, determined using less concentrations of the radioligand with the corresponding 95% confidence intervals (**b**). Data are expressed as mean ± SEM from individual experiments (*n* ≥ 4)

Kinetic real-time cell-binding experiments were performed to determine the *K*_*d*_ of the radiolabelled A_3_R agonist [^125^I]-AB-MECA based on the quantification of the rate constants *k*_on_ and *k*_off_. The observed association time-courses of the dedicated radioligand concentrations were obtained with high spatial and temporal resolution (Fig. 6a). Applied curve fitting algorithms were proved to be consistent with the measured data points, and best-fit estimations for *k*_on_ and *k*_off_ were determined using Eqs. (5) and (6) with high reliability and accuracy (Table 2). To validate these findings, the binding traces of [^125^I]-AB-MECA were analysed separately from each other using one-phase association curve fitting algorithm and resulting *k*_obs_ values were plotted against the dedicated radioligand concentration (Fig. 6b). Linear regression analysis was found to show high correlation (*R* ^2^ = 0.9927) and revealed values for *k*_on_ of 8.859 × 10^7^ ± 0.299 × 10^7^ M^−1^ min^−1^ and for *k*_off_of 0.049 ± 0.006 min^−1^, which are in good agreement with the rate constants obtained from the global fitting approach. To control confounding factors due to potential variations of the dedicated target protein, an assessment of associated hA_3_R expression levels was obtained using Eq. (7). Corresponding signal values were found to confirm homogenous receptor expression within the whole binding experiment (Fig. 6c). The difference between the *K*_*d*_ of [^125^I]-AB-MECA obtained from our kinetic binding approach and the binding data as previously reported in the literature with traditional saturation binding assays on membranes from human A_3_R-transfected HEK-293 cells [29] was proved to be statistically not significant (*P* > 0.05) using two-tailed, unpaired Student’s *t* test with Welch’s correction (Fig. 6d). A detailed summary of *k*_on_, *k*_off_ and *K*_*d*_ values are given in Table 2. Fitting parameters for *k*_on_ and *k*_off_ were obtained using a set of eight different concentrations of [^125^I]-AB-MECA spanning two orders of magnitude needed for proper affinity estimation. Reliability and effectiveness of the experimental approach were proved by comparing the resulting *K*_*d*,_ determined using the full set of concentrations, with the corresponding *K*_*d*_ values, calculated from best-fit estimations for the rate constants using two, three, four, five, six or seven concentrations of the radioligand, respectively. Differences between *K*_*d*_ values were determined to be statistically not significant (*P* > 0.05), using ordinary one-way ANOVA with Tukey’s multiple comparisons correction (Fig. 7a). Best-fit estimations were found to be less precise when using only a set of two, three or four concentrations of radioligand. An analysis of the differences between averaged *K*_*d*_ values revealed that there is a high preservation of the estimated binding affinity, starting from the usage of five radioligand concentrations (Fig. 7b).

**Table 2:** Best-fit estimations of the rate constants and the resulting *K*_*d*_ values in kinetic real-time cell-binding experiments

Fitting approach	*k*_on_ [M^−1^ min^−1^]	*k*_off_ [min^−1^]	*K*_*d*_ [nM]	*R* ^2^	*df*
2 concentrations	8.675 × 10^7^ ± 0.414 × 10^7^	0.077 ± 0.008	0.887 ± 0.121	0.992	216
3 concentrations	9.262 × 10^7^ ± 0.835 × 10^7^	0.049 ± 0.007	0.528 ± 0.118	0.991	325
4 concentrations	9.243 × 10^7^ ± 0.919 × 10^7^	0.051 ± 0.007	0.556 ± 0.132	0.988	434
5 concentrations	8.805 × 10^7^ ± 0.289 × 10^7^	0.062 ± 0.003	0.704 ± 0.054	0.994	543
6 concentrations	8.788 × 10^7^ ± 0.292 × 10^7^	0.061 ± 0.003	0.695 ± 0.052	0.993	652
7 concentrations	8.105 × 10^7^ ± 0.259 × 10^7^	0.055 ± 0.003	0.683 ± 0.056	0.993	761
8 concentrations	8.068 × 10^7^ ± 0.260 × 10^7^	0.057 ± 0.003	0.711 ± 0.056	0.993	870
Averaged	6.645 × 10^7^ ± 1.423 × 10^7^	0.043 ± 0.014	0.636 ± 0.076	nd	nd
Reported affinity	nd	nd	0.59 ± 0.02 [29]	nd	nd

Curve fitting parameters of the association rate constant and the dissociation rate constant of [^125^I]-AB-MECA on CHO-K1-hA3R cells at ambient temperature. Dedicated *K*_*d*_ values were calculated as the ratio of *k*_off_/*k*_on_. The table summarizes best-fit estimations and corresponding goodness of fit parameters for a set of different amount of concentrations of [^125^I]-AB-MECA. The “averaged” value comprises data from three independent experiments. Reported *K*_*d*_ was determined with traditional saturation binding assays using membranes from human A_3_R-transfected HEK-293 cells. Data in the table are expressed as mean ± SEM from individual experiments (*n* ≥ 4)

*nd* not determined

## Discussion

Radioligand-binding studies provide excellent quantitative data with high sensitivity, but are time-consuming, laborious and lack the possibility of high spatial and temporal data acquisition [16]. Considering this, the described approaches improve the determination of binding kinetics, resulting in a more time-efficient and comprehensive data acquisition, with high spatial and temporal resolution. Resolving the kinetic mechanisms of biomolecular interactions has become more and more important to improve the performance of binding experiments and to avoid systematic bias and potential errors of obtained data under equilibrium and non-equilibrium conditions [5–7]. This consideration is supported by an increasing amount of studies, which address a shift from traditional approaches to the characterization of binding kinetics [5, 6, 10–16, 22, 23]. In the present study, we successfully designed and established two experimental approaches for the reliable in vitro assessment of binding affinity of both, radiolabelled and non-labelled compounds, based on high-resolution real-time data acquisition of radioligand-receptor binding kinetics. Realized as competitive real-time cell-binding studies, we introduced an accurate method for the visualization and quantification of radioligand dissociation in the presence of different non-labelled competitor concentrations (Fig. 4). Time-resolved quantitative data about the kinetic mechanisms of the competition, provide information beyond the fact that competition occurs, including the relative binding strength or the affinity of the competitor itself. We determined in our competitive real-time cell-binding studies the *K*_*i*_ of eight different A_3_R antagonists with high reliability (Table 1) and highlighted the suitability of the used method as we confirmed previously published *K*_*i*_ values of MRS1191, MRS1523 and FE@SUPPY (Fig. 5). In addition, we demonstrated that the experimental performance was not confounded by variations of the lipophilicity of the tested compounds. In contrast to in vitro binding experiments, in an in vivo setting, the concentration of a dedicated ligand to its target region changes over time and is often influenced by additional factors other than basic biomolecular ligand-receptor interactions. As a consequence, the in vitro measured *K*_*d*_ alone provides not sufficient information to characterize complex compound interactions and the potential in vivo effectiveness of small-molecule drugs, but rather the in vitro assessment of radioligand-receptor binding kinetics. Furthermore, we successfully established in kinetic real-time cell-binding studies an experimental design for the effective and reliable quantification of the rate constants *k*_on_ and *k*_off_ in order to determine the *K*_*d*_ of a radiolabelled ligand. Binding experiments with the radiotracer [^125^I]-AB-MECA, a potent A_3_R agonist, were found to reveal information of the observed association time courses at various concentrations with high spatial and temporal resolution (Fig. 6). Thus, we determined best-fit estimations for *k*_on_ and *k*_off_ with high reliability and precision, and the resulting *K*_*d*_ was proved to be in good agreement with the affinity previously reported with traditional saturation binding approaches (Table 2; Fig. 6d). In the performance analysis of the experimental approach, we found that, even with a low number of radioligand concentrations, an appropriate estimation of the dedicated *K*_*d*_ was possible, making it suitable for cost-efficient screening of potential drug candidates. Moreover, we obtained an estimation of the associated hA_3_R expression levels in order to control confounding factors due to potential variations of the dedicated target protein density (6C).

## Conclusions

In conclusion, we found that, compared to traditional in vitro binding assays, the time-resolved kinetic approaches described in this paper have several advantages and the practical use is potentially wide. When adding either the radioligand or the competitor, a steady insight into association, retention and dissociation of the radiolabelled ligand is possible during the experiment with high spatial and temporal resolution. This information can be used for the exact determination of binding equilibrium and provides an increased understanding of the occasional complex interplay between radiotracer, ligand and the dedicated biological target. Moreover, it has the potential to facilitate the experimental workflow as it enables interactive intervention, minimizes the quantities of the used radiotracer and reduces systematic errors caused by non-equilibrium conditions. In addition, we found that continuously acquired time-resolved kinetic data help to improve the computational postprocessing by providing refined statistical accuracy and advance the formulation of computational methods to analyse biomolecular interactions of ligands with the receptor alone, or in combination. Both kinetic binding approaches comprise tracer administration and subsequent binding to living cells, expressing the dedicated target protein. Therefore, the experiments are more similar to the in vivo physiological conditions and provide important markers of cellular feedback and biological response. Taken all together, the reliable in vitro assessment of time-resolved radioligand-receptor binding kinetics has the potential to provide comprehensive information to the development of radiolabeled probes for molecular imaging.

## Abbreviations

A_3_R: Adenosine A3 receptor
*B*_Max_: Concentration of specific binding sites
CHO-K1: Chinese hamster ovary cells
CHO-K1-hA_3_R: Chinese Hamster ovary cells stably expressing the human adenosine A3 receptor
FBS: Fetal bovine serum
G-418: Geneticin
GPCR: G-protein-coupled receptor
_HPLC_logD^7.4^: Logarithm of the octanol-water partition coefficient
IC_50_: Half-maximum inhibitory concentration
K_d_: Equilibrium dissociation constant
K_i_: Equilibrium inhibitory constant
k_obs_: Observed rate constant of the association reaction
k_off_: Dissociation rate constant
*k*_on_: Association rate constant
PSG: Penicillin-streptomycin-glutamine
